# Automatic Quantification Software for Geographic Atrophy Associated with Age-Related Macular Degeneration: A Validation Study

**DOI:** 10.1155/2020/8204641

**Published:** 2020-08-05

**Authors:** José M. Ruiz-Moreno, Jorge Ruiz-Medrano, Francisco Lugo, Belen Sirvent, Ignacio Flores-Moreno

**Affiliations:** ^1^Department of Ophthalmology, Castilla-La Mancha University, Albacete, Spain; ^2^Puerta de Hierro-Majadahonda University Hospital, Madrid, Spain; ^3^Vissum Corporation, Alicante, Spain

## Abstract

**Aims:**

To determine the accuracy and repeatability of new software to automatically quantify GA areas associated to age-related macular degeneration (AMD) by swept-source optical coherence tomography (SS-OCT). *Settings and Design*. Tertiary referral hospital in Spain. Cross-sectional and noninterventional. *Methods and Material*. Forty-six eyes from 33 AMD patients with GA, without previous choroidal neovascularization, were scanned using a SS-OCT (Topcon Corporation, Japan), including three consecutive 7 × 7 mm OCT scans. Three independent masked observers manually measured the GA area using FAF images. These measures were compared to the three automatic determinations of the GA. Lesions were classified according to their morphology and number as regular/irregular and single/multiple. *Statistical Analysis Used*. Intraclass correlation coefficients (ICCs) were estimated to study the agreement between the three physicians in manual measurements. ICC through a two-way mixed effects model was used for the software measures, and Lin's concordance correlation coefficient (CCC) was used to analyse the agreement between the physicians and the software.

**Results:**

The mean age was 76.3 ± 11.7 years. Eighteen cases showed regular lesions, and 30 showed single lesions. The CCC between manual and automatic measures was 0.95 for the whole sample. The CCC for the area according to the lesion type was 0.92 and 0.97; it was 0.99 for single lesions and 0.89 for multiple lesions. The ICC between the three physicians was 0.94 for the whole sample and 0.88 in multiple lesions. The ICC between the three automatic measures for the area was 0.98 for the whole sample, regular or irregular lesions, and single or multiple lesions.

**Conclusions:**

The accuracy of this new software is substantial for the area with a high degree of repeatability agreement, being very precise in single lesions.

## 1. Introduction

The International Age-Related Maculopathy Epidemiological Study Group defined geographic atrophy (GA) in 1995 using colour fundus photography (CFP) as “any sharply delineated roughly round or oval area of hypopigmentation or depigmentation with increased visibility of the underlying choroidal vessels and of at least 175 *μ*m in diameter on 30° or 35° CFP images” [1]. GA is a well-established end-stage manifestation of age-related macular degeneration (AMD) [2, 3]. Gass originally described “geographic areas of atrophy” in the context of “senile macular choroidal degeneration” in 1970 [4, 5].

However, GA limits are not easily identified using monoscopic images, so high-quality, stereoscopic imaging is often needed in order to adequately delineate these lesions, with the latter being difficult to obtain in certain cases. There has recently been a switch from this technology to fundus autofluorescence (FAF) imaging as the main tool to detect, monitor, and quantify GA lesions [6, 7], which appear well-delineated using blue or green light FAF [8]. This high contrast between a healthy and atrophic retinal pigment epithelium (RPE) has provided ophthalmologists with a reproducible, reliable method to analyse and manually quantify GA, having been used in several clinical trials [9, 10]. Nevertheless, manual determinations are time-consuming and can be affected by the observer's subjectivity.

One downside of blue light FAF for the identification of GA lesions is the fact that the luteal pigment of the central macula absorbs blue excitation light, which makes foveal evaluation a difficult task [11]. In order to avoid this problem, blue light FAF adds near-infrared reflectance, which is not affected by the luteal pigment, allowing a correct study of the fovea.

Optical coherence tomography (OCT) has recently become a fundamental tool for macular evaluation [12, 13]. The high axial resolution of the latest Fourier-domain devices (both spectral-domain and Swept-Source OCT (SS-OCT)) allow for a three-dimension study of atrophic areas, detecting affection and tissue loss of every single retinal layer. Moreover, conventional B-scans together with volumetric OCT exams help towards achieving a better identification of GA borders.

The latest, high-resolution OCT devices give us the opportunity to identify the earliest phases of this disease (nascent atrophic AMD), even before lesions are clinically visible or before the use of CFP and FAF [14]. In addition, OCT provides a layer-by-layer tissue analysis, which is crucial as atrophic AMD-related cell loss may vary between layers. This technology can offer the possibility to quantify GA based on OCT-defined changes using different retinal and choroidal layers as reference as the atrophy advances.

Although GA growth can only be defined using CFP or FAF (per clinical trial regulation agencies), the use of OCT could potentially provide an earlier, more accurate detection of changes related to tissue loss [15].

The aim of this paper is to determine the accuracy and repeatability of the new software in automatically quantifying the GA area associated with AMD using swept-source optical coherence tomography (SS-OCT).

## 2. Subjects and Methods

**Figure 1:**
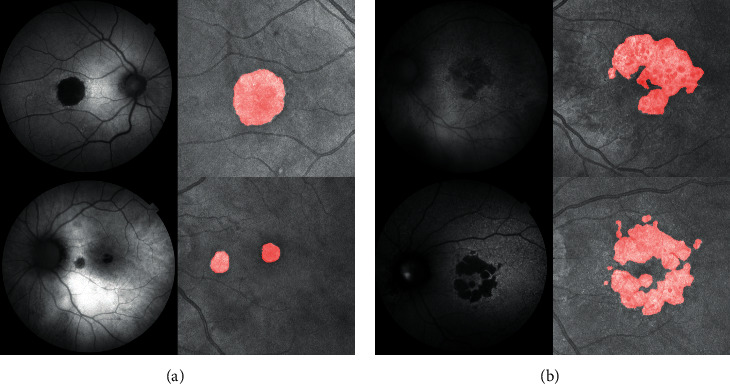
Examples of different patients suffering from regular (a) and irregular (b) atrophy in the context of age-related macular degeneration.

This was a cross-sectional, noninterventional study. It followed the tenets of the Declaration of Helsinki. Forty-six eyes from 33 AMD patients with GA, without previous choroidal neovascularization were examined using a Triton Swept-Source OCT (Topcon Corporation, Japan). The OCT study included CFP, FAF, and 7 × 7 mm OCT cubes using eye tracking (three consecutive times). The lesions were classified according to their morphology and number as regular/irregular and single/multiple (Figure 1). Three independent observers performed the manual measurement of the area of the GA lesion at a FAF image in a masked fashion. These manual measures of the area were compared to the three automatic determinations of the area of GA performed in each of the SS-OCT cubes obtained.

**Figure 2:**
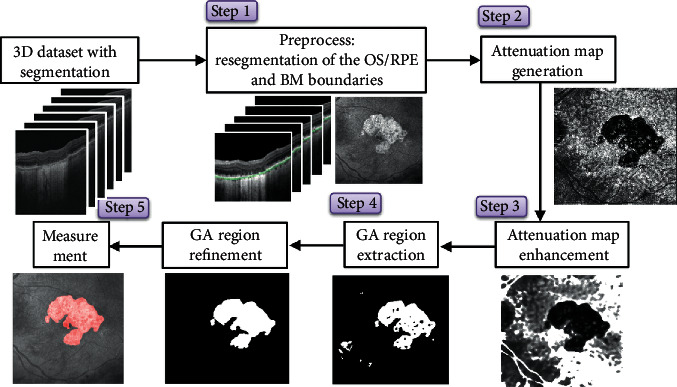
The geographic atrophy (GA) analysis algorithm workflow consists of five steps: preprocessing of the data and layer segmentation results, generation of the integrated attenuation map, enhancement of the attenuation map, extraction of the GA region, and postprocessing of the results. The preprocessing step includes resegmentation of the outer segments/retinal pigment epithelium and Bruch's membrane boundaries in each B-scan for better detection in typical GA patients and the disc mask generation in the *X*-*Y* plane from the input fovea and disc locations.

The GA module is primarily based on an integrated attenuation program, in which the signal intensities of the RPE complex are normalized by the choroid-scleral signal as part of the calculation. This method, patented GA detection methodology (US2016/0206190), is an objective measure of physical tissue characteristics and is less sensitive to imaging conditions, such as variations in opacities and various types of signal shadowing, compared to the pure intensity integration method. Using this method, a 7 × 7 mm cube scan centred at the macula was captured by using a DRI OCT Triton. The GA module operation starts with a 3D volume scan and provides the GA region map and area calculation in the *X*-*Y* plane. The GA analysis algorithm workflow consists of the following five steps: preprocessing of the data and layer segmentation results, generation of integrated attenuation map, enhancement of the attenuation map, extraction of the GA region, and postprocessing of the results. The preprocessing step includes resegmentation of the outer segments/RPE and Bruch membrane boundaries in each B-scan for better detection in typical GA patients and the disc mask generation in the *X*-*Y* plane from the input fovea and disc locations. The integrated attenuation map was generated using the ratio of the signal intensities of the RPE complex over the choroid-scleral signal. The attenuation map enhancement employs various noise reduction techniques, including order filtering. The GA region extraction is, then, performed by smart thresholding and refinement steps. The postprocessing step confirms the detected GA region using a sub-RPE map and adjusts the region if necessary (Figure 2).

### 2.1. Statistical Analysis

Quantitative data were described as the mean, standard deviation, and range, while qualitative data were described as the frequency distribution. Intraclass correlation coefficients (ICCs) were estimated through a two-way random effects model with (i) a corresponding 95% confidence interval (CI) to study the agreement between the three physicians in manual measurement in FAF (interobserver agreement); (ii) the ICC through a two-way mixed effects model and 95% CI to study the agreement between three software consecutive measures (intraobserver agreement); and (iii) Lin's concordance correlation coefficient (CCC) to analyse the agreement between the three average manual physicians measures and the average of the three automatic software measures.

We considered the following limits of agreement for CCC < 0.90: poor; 0.90 to 0.95: moderate; 0.95 to 0.99: substantial; and >0.99 almost perfect.

All analyses were performed for the whole sample and repeated according to lesion type (regular or irregular) and the number of lesions (single or multiple).

Statistical significance was defined as *p* < 0.05. The statistical analysis was performed using Stata v15.1 (StataCorp. 2017. *Stata Statistical Software: Release 15*. College Station, TX: StataCorp LLC), and the CONCORD module was used to estimate the concordance correlation coefficient (CCC); (Nicholas J. Cox & Thomas Steichen, 2000. “CONCORD: Stata module for concordance correlation,” Statistical Software Components S404501, Boston College Department of Economics) [16].

## 3. Results

The mean age of our patients was 76.3 ± 11.7 years (from 54 to 96); and 20 were males. Eighteen had regular lesions, and 30 cases showed one single lesion (Table 1).

**Figure 3:**
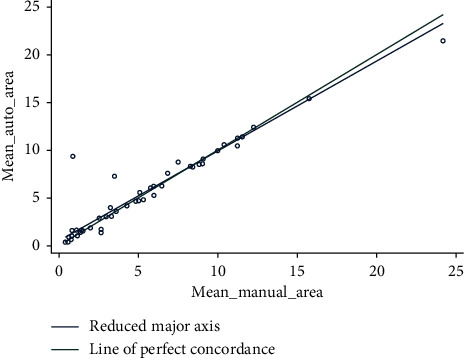
Lin's concordance correlation coefficient to analyse the agreement between the three average manual measures and the mean of three automatic software measures was 0.95 (95% CI 0.93/0.96) for the whole sample.

The CCC to analyse the agreement between the three average manual measures and the mean of three automatic software measures was 0.95 (95% CI 0.93/0.96) for the whole sample (Figure 3).

**Figure 4:**
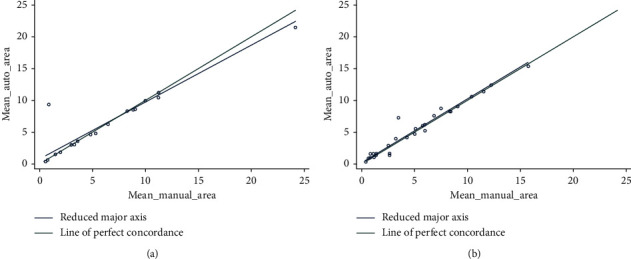
Lin's concordance correlation coefficient for the area according to the lesion type was moderate (0.92) and substantial (0.97) for regular (a) and irregular lesions (b), respectively.

**Figure 5:**
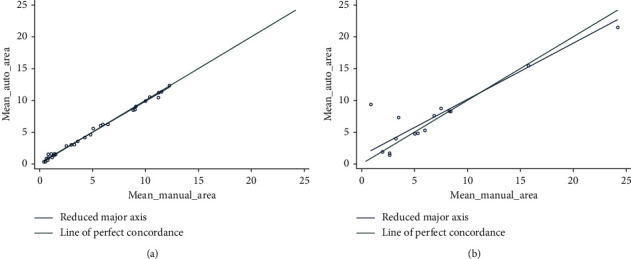
Lin's concordance correlation coefficient was almost perfect (0.99) for single lesions (a) being poor for multiple lesions (0.89) (b).

The CCC for the area according to the lesion type was moderate (0.92) and substantial (0.97) for regular and irregular lesions, respectively (Figure 4), and almost perfect (0.99) for single lesions, being poor for multiple lesions (0.89) (Figure 5) (Table 2).

The ICC to study the agreement between the three physicians' manual measurements using FAF (interobserver agreement) was moderate for the whole sample measures (0.94). The ICC was poor (0.88) in multiple lesions (Table 3).

The ICC to study the agreement between the three automatic consecutive measures (intraobserver agreement) for the area was substantial (0.98) for the whole sample, regular or irregular lesions, and single or multiple lesions (Table 4). Automatic measurements for both the area and perimeter were larger than manual measurements, although differences did not reach statistical significance (Table 5).

## 4. Discussion

GA is a clinical manifestation of advanced AMD. The need and the importance of developing software that automatically quantifies GA lesions' areas has been thoroughly discussed [17–21]. Interventionist clinical trials targeting atrophic AMD are the best example of the need for a precise monitoring tool that could help quantify the progression rate of this disease.

FAF images clearly show the borders of these lesions and are currently accepted as the gold-standard to evaluate the progressive growth of GA. However, as mentioned above, SD and SS-OCT show different microstructural alterations in relation to GA which cannot be detected using FAF [20]. Furthermore, it is a method both time-consuming and prone to intra- and interobserver variability, which stresses the need for automated methods to automatically determine these areas, establish progression, and help the clinical diagnosis [21–23]. To back this tendency, the CAM group defends the use of OCT and has recently proposed a new OCT-based classification for atrophic AMD. They introduced four new terms: complete RPE and outer retinal atrophy (cRORA), incomplete RPE and outer retinal atrophy (iRORA), complete outer retinal atrophy, and incomplete outer retinal atrophy [24].

Reports discussing some of the advantages of OCT for the study of GA in comparison to FAF have already been released. Sayegh et al. compared SD-OCT to confocal scanning-laser ophthalmoscopy (cSLO) FAF for the classification of GA and reached the conclusion that hypofluorescent lesions were linked to RPE loss and outer retinal layer alterations detected with SD-OCT. The lesion size could be determined with accuracy, and foveal affection was more precise when using OCT [25]. Schmitz-Valckenberg et al. observed that progressive photoreceptor loss with outer retinal thinning could happen in some cases with a healthy RPE. These cases could only be detected using OCT and not with classic FAF [6].

Several authors published their results on semiautomatic quantification of GA using FAF imaging. Pfau et al. compared the agreement between observers and imaging modalities (means/methods) using green (GAF; 518 nm) vs. combined blue (BAF; 488 nm) and near-infrared light (NIR; 820 nm). The ICC using GAF was 0.995, followed by BAF + NIR with 0.992 and a BAF alone with 0.991 [26]. Panthier et al. analysed the intra- and interobserver agreement using a novel FAF semiautomatic software for the quantification of GA, using Bland–Altman statistic analysis, concluding that their software is a reliable tool to identify and quantify GA [27]. Schmitz-Valckenberg et al. studied intra- and interobserver longitudinal measurement variability using semiautomated, FAF-based GA quantification software and determined that it was an accurate, reproducible, and time-efficient technique [9].

Our ICC results regarding the agreement between the three physicians' manual measurements using FAF was 0.94 for the whole sample for area measures, showing worse concordance (0.88) in the analysis in cases of multiple lesions.

There are also some reports on automatic determinations of GA using FAF and OCT. Ramsey et al. developed a computerized segmentation method seeking to standardize the quantification of GA and compared its results to manual measuring in 10 patients, reaching a sensitivity of 94 ± 5% and a specificity of 98 ± 2% for the detection of GA [28].

Lee et al. automatically quantified GA using 100 FAF images, showing a mean sensitivity/specificity of 98.3/97.7% manually vs. 88.2/96.6% using their algorithm [21]. The same group described a hybrid segmentation method to quantify GA via the identification of hypofluorescent regions on FAF, with a mean sensitivity and specificity of 0.89  and 0.98% in ROC analysis [23]. Bauman et al. used polarization-sensitive OCT to quantify atrophic areas of dry AMD patients, calculating their dimensions, concluding that it may be a promising modality [29].

In our series, the CCC reached when analysing the agreement between manual and automatic software measures was substantial (0.95) for the whole sample. The CCC for the area regarding each lesion type was moderate (0.92) and substantial (0.97) for regular and irregular lesions, respectively, and almost perfect (0.99) for single lesions, being poor for multiple lesions (0.89). Software repeatability threw very good results, with ICC values between the three automatic consecutive measures being substantial (0.98) for the area, regardless of the shape or the number of the lesions. Additionally, this new software seems to pick up changes faster than FAF measurements, despite not reaching statistical significance.

There were only two previous studies using OCT-based automatic analyses of GA areas. Chen et al. described a semiautomated segmentation algorithm based on SD-OCT and compared it to FAF obtaining an accuracy of 72.60% [30]. This last group developed a segmentation system for SD-OCT, using a Chan–Vese model and compared it to manual segmentation. The algorithm showed an OR of 81.86% and 70%, respectively [17]. Our series' results cannot be compared with these two studies, as the statistical analyses performed were different.

This study shows several limitations. There is no other accurate system to compare our results to, as although manual FAF measures are accepted, they are always limited by observer variability. Other limitations include the sample size and the subjective classification of lesions into regular or irregular.

In conclusion, this is new, fully-automatic GA detection software that allows a fast, objective, and reliable analysis of GA. Its accuracy is substantial for the area with a high degree of repeatability agreement, being particularly precise in single lesions. Its segmentation in cases with multiple lesions must be improved (although manual graders also demonstrated poor repeatability in these cases). These results should be checked over a larger series of patients with GA.

## Figures and Tables

**Table 1:** Demographics.

Lesions	Regular	Irregular	Total
Single	13	17	30
Multiple	5	11	16
Total	18	28	46

**Table 2:** Agreement between manual vs. automatic area measurements.

Sample	Group	CCC	95% CI	Degree of agreement
Whole		0.95	0.93 0.96	Substantial

Lesion type	1	0.92	0.88 0.96	Moderate
	2	0.97	0.96 0.98	Substantial

Number of lesions	1	0.99	0.99 0.99	Almost perfect
	2	0.89	0.83 0.94	Poor

CCC: concordance correlation coefficient. CI: confidence interval. Lesion type- 1: regular; 2: irregular. Number of lesions- 1: single; 2: multiple.

**Table 3:** Interobserver agreement (area).

Sample	Group	ICC	95% CI	Degree of agreement
Whole		0.94	0.89 0.96	Moderate

Lesion type	1	0.89	0.77 0.96	Poor
	2	0.99	0.98 0.99	Almost perfect

Number of lesions	1	0.99	0.98 0.99	Almost perfect
	2	0.88	0.74 0.96	Poor

ICC : intraclass correlation coefficient. CI: confidence interval. Lesion type- 1: regular; 2: irregular. Number of lesions- 1: single; 2: multiple.

**Table 4:** Intrasoftware agreement (area).

Sample		Group	ICC	95% CI	Degree of agreement
Whole			0.98	0.97 0.99	Substantial

Lesion type		1	0.98	0.96 0.99	Substantial
		2	0.98	0.97 0.99	Substantial

Number of lesions		1	0.98	0.96 0.99	Substantial
		2	0.98	0.95 0.99	Substantial

ICC: intraclass correlation coefficient. CI: confidence interval. Lesion type- 1: regular; 2: irregular. Number of lesions- 1: single; 2: multiple.

**Table 5:** Area and perimeter values.

Variable	Automatic	Manual	*p* value	Statistical test
Area	5.81 ± 4.5	5.63 ± 4.7	0.69	Mann–Witney *U* test
	0.35–21.47	0.39–24.18		

Perimeter	19.08 ± 11.6	16.75 ± 9.3	0.47	Mann–Witney *U* test
	3.27–48.88	3.58–37.36		

## Data Availability

The data used to support the findings of this study are available from the corresponding author upon request.
